# Correlation of somatostatin receptor PET/CT imaging features and immunohistochemistry in neuroendocrine tumors of the lung: a retrospective observational study

**DOI:** 10.1007/s00259-022-05848-z

**Published:** 2022-06-08

**Authors:** Vittoria Rufini, Margherita Lorusso, Frediano Inzani, Tina Pasciuto, Elizabeth Katherine Anna Triumbari, Lucia Rosalba Grillo, Filippo Locco, Stefano Margaritora, Edoardo Pescarmona, Guido Rindi

**Affiliations:** 1 Section of Nuclear Medicine, University Department of Radiological Sciences and Hematology, Università Cattolica del Sacro Cuore, Rome, Italy; 2 Unit of Nuclear Medicine, Fondazione Policlinico Universitario A. Gemelli IRCCS, Rome, Italy; 3 ENETS Center of Excellence for the Diagnosis and Cure of Neuroendocrine Tumors, Rome, Italy; 4 PET/CT Center, Fondazione Policlinico Universitario Agostino Gemelli IRCCS, Rome, Italy; 5 Unit of Pathology, Department of Woman and Child Health and Public Health, Fondazione Policlinico Universitario A. Gemelli IRCCS, Rome, Italy; 6 Research Core Facility Data Collection G-STeP, Fondazione Policlinico Universitario A. Gemelli IRCCS, Rome, Italy; 7 Pathology Unit, San Camillo-Forlanini Hospitals, Rome, Italy; 8 Section of Thoracic Surgery, Department of Translational Medicine and Surgery, Università Cattolica del Sacro Cuore, Rome, Italy; 9 Unit of Thoracic Surgery, Fondazione Policlinico Universitario A. Gemelli IRCCS, Rome, Italy; 10 Pathology Unit, ‘Regina Elena’ National Cancer Institute IRCCS, Rome, Italy; 11 Section of Pathology, Department of Woman and Child Health and Public Health, Università Cattolica del Sacro Cuore, Rome, Italy

**Keywords:** Lung NET, [^68^Ga]-DOTA-somatostatin analogs, PET/CT, Somatostatin receptor subtypes, Ki-67, Immunohistochemistry

## Abstract

**Purpose:**

To correlate somatostatin receptor (SSTR) and proliferative activity profile (SSTR2, SSTR5, Ki-67) at immunohistochemistry (IHC) with SSTR-PET/CT imaging features in a retrospective series of lung neuroendocrine tumors (NET). Proliferative activity by Ki-67 and ^18^F-FDG-PET/CT parameters (when available) were also correlated.

**Methods:**

Among 551 patients who underwent SSTR-PET/CT with ^68^Ga-DOTA-somatostatin analogs (SSA) between July 2011 and March 2020 for lung neuroendocrine neoplasms, 32 patients with a confirmed diagnosis of NET were included. For 14 of them, ^18^F-FDG-PET/CT was available. PET/CT images were reviewed by qualitative and semi-quantitative analyses. Immunohistochemistry for SSTR2, SSTR5, and Ki-67 was assessed. Inferential analysis was performed including kappa statistics and Spearman’s rank correlation test.

**Results:**

Definitive diagnosis consisted of 26 typical carcinoids-G1 and six atypical carcinoids-G2. Positive SSTR2-IHC was found in 62.5% of samples while SSTR5-IHC positivity was 19.4%. A correlation between SSTR2-IHC and SSTR-PET/CT was found in 24/32 cases (75.0%, *p* = 0.003): 20 were concordantly positive, 4 concordantly negative. For positive IHC, 100% concordance with SSTR-PET/CT (both positive) was observed, while for negative IHC concordance (both negative) was 33.3%. In 8 cases, IHC was negative while SSTR-PET/CT was positive, even though with low-grade uptake in all but one. A significant correlation between SUV_max_ values at SSTR-PET/CT and the SSTR2-IHC scores was found, with low SUV_max_ values corresponding to negative IHC and higher SUV_max_ values to positive IHC (*p* = 0.002).

**Conclusion:**

This retrospective study showed an overall good agreement between SSTR2-IHC and tumor uptake at SSTR-PET/CT in lung NETs. SSTR-PET/CT SUV_max_ values can be used as a parameter of SSTR2 density. Within the limits imposed by the relatively small cohort, our data suggest that SSTR2-IHC may surrogate SSTR-PET/CT in selected lung NET patients for clinical decision making when SSTR-PET/CT is not available.

## Introduction

Neuroendocrine neoplasms (NEN) of the lung represent 20–30% of all NEN and about 20% of all lung malignancies [1]. According to the recent World Health Organization (WHO) – International Agency for the Research on Cancer proposal for a standardized NEN nomenclature and the current WHO classification, typical and atypical lung carcinoids (TC and AC) belong to the well-differentiated neuroendocrine tumor (NET) family and are graded as G1 and G2, respectively, while the small cell lung cancer (SCLC) and the large cell neuroendocrine carcinoma (LCNEC) are poorly differentiated neuroendocrine carcinomas (NEC) by default of grade 3 [2, 3]. SCLC is by far the largest fraction of lung NEN accounting for 15% of lung cancer; minor fractions are LCNEC accounting for only 3% of resected lung cancers as well as TC and AC, which account for ≤ 2% of all lung malignancies with prevalent TC vs AC [4].

Like NETs of other anatomical sites, most lung NETs express somatostatin receptors (SSTRs), mainly the subtype 2 (SSTR2) [5]. This feature is the basis for the widespread application of diagnostic and therapeutic strategies with “cold” or radiolabeled somatostatin analogs (SSA) in NETs [6, 7]. SSTR-positron emission tomography/computed tomography (SSTR-PET/CT) with ^68^Ga-labeled-SSA is currently used in lung TC-AC/NET for in vivo SSTR characterization and staging [8, 9]. ^18^F-FDG PET/CT, which is the elective functional imaging modality for the highly proliferative SCLC and LCNEC [10], has also been explored in lung TC-AC/NET and proved to correlate with cancer cell proliferation [11, 12].

In clinical routine, in vitro SSTR expression can be detected on tissue samples by immunohistochemistry (IHC), a reproducible and sensitive routine procedure, which provides information on cellular/subcellular SSTR subtype distribution in tumor cells [13]. An alternative method is reverse transcriptase-polymerase chain reaction (RT-PCR), which demonstrates SSTR mRNA [14]; however, its use is hampered in pathology services by limited access to molecular technology with relative complexity and high costs as compared to the widespread access, robustness, and low cost of IHC. The correlation of SSTR2-IHC expression vs functional imaging data (in vivo expression) has been assessed in mixed series of patients with various NET types by using SSTR-scintigraphy (Octreoscan®) or ^68^Ga-DOTATOC PET/CT [13, 15]. Limited data are available for lung NET [5].

The aim of this study was to assess the somatostatin receptor and proliferative activity profile (SSTR2, SSTR5, Ki-67) at IHC in a retrospective selected series of lung NET, to correlate it with SSTR-PET/CT imaging features, and to specifically assess the potential role of IHC in predicting in vivo SSTR expression. Secondly, we aimed to correlate proliferative activity by Ki-67 with ^18^F-FDG-PET/CT parameters (when available) in the same cohort.

## Materials and methods

### Study population

All consecutive patients with a suspected lung NEN who underwent SSTR-PET/CT at our Centre between July 2011 and March 2020 were considered for analysis. Inclusion criteria were SSTR-PET/CT performed for radiological or cytological suspicion of lung NEN; no previous treatment (surgery, chemo- or radiotherapy or “cold” somatostatin analogues); confirmed histopathological diagnosis of TC-AC lung NET by lesion biopsy or after surgery; and availability of tissue material for pathological review and immunohistochemistry. When available, ^18^F-FDG-PET/CT studies performed within a 2-month period from SSTR-PET/CT were also reviewed.

All PET/CT studies were performed in the routine clinical practice, for functional characterization of tumor lesions and/or disease staging. This retrospective observational study was approved by the local Ethics Committee (protocol n. 16162/13), and a written informed consent was obtained from all individual participants included in the study.

### PET/CT protocols

PET/CT was performed on a hybrid scanner (Gemini GXL, Philips Medical Systems, Cleveland, Ohio; or Biograph mCT, Siemens Medical Solution, Erlangen, Germany). Low-dose CT scan (120 keV, 80 mA tube current) was acquired for anatomical localization and attenuation correction. For the Siemens Biograph mCT, 3D OSEM reconstruction with PSF modeling/TOF (2 iterations and 21 subsets, voxel size of 3.2 × 3.2 × 5 mm3) was applied. For the Philips Gemini GXL, LOR RAMLA reconstruction (2 iterations and 24 subsets, voxel size: 4 × 4 × 4 mm^3^) was applied.

#### ^68^Ga-DOTA-SSA-PET/CT protocol

Throughout the study period, both ^68^Ga-DOTANOC (from July 2011 to April 2015) and ^68^Ga-DOTATOC (from May 2015 onwards) were used. ^68^Ga was obtained from a ^68^Ge/^68^Ga generator (IGG 100; Eckert & Ziegler Isotope Products, Berlin, Germany), with a nominal activity of 1.85 GBq. ^68^Ga-DOTANOC was produced according to a previously described protocol [16] whereas ^68^Ga-DOTATOC was produced according to the European Pharmacopoeia monograph [17]. PET/CT scans were performed at 45 ± 10 min after intravenous administration of ^68^Ga-DOTA-peptides (2 MBq/kg). CT and PET images were acquired from vertex to mid-thigh (4 min/bed position).

#### ^18^F-FDG-PET/CT protocol

^18^F-FDG-PET/CT was performed after at least 5 h of fasting. PET images were acquired 60 ± 10 min after intravenous injection of ^18^F-FDG (3.7 MBq/kg). At the time of tracer injection, all patients presented blood glucose levels < 200 mg/dl and were hydrated with 500 ml of saline solution. CT and PET images were recorded from skull base to mid-thigh (3 min/bed position).

### PET/CT imaging interpretation, data collection, and analysis

PET/CT images were independently reviewed by 2 nuclear medicine physicians (M.L. and E.K.A.T.) blinded to clinical reports, who reached a consensus; any disagreements were solved by a third senior reviewer (V.R.). Both qualitative and semi-quantitative analyses were performed. For qualitative analysis, any focal tracer uptake in the lung nodule higher than the surrounding physiologic uptake was considered an abnormal (positive) finding. The detection rate for both tracers was determined by the number of positive studies and the number of patients with lung NEN, according to histological diagnosis. For the visual assessment of SSTR-PET/CT images, the Krenning score, a visual scoring method usually used to assess the degree of tracer uptake on ^111^In-pentetreotide scintigraphy, was also applied [18]. According to this score, abnormal uptake was graded from 0 to 4 by using liver and spleen as reference organs: 0 = no uptake; 1 = uptake lower than liver; 2 = uptake slightly less than or equal to liver; 3 = uptake greater than liver; 4 = uptake greater than spleen. Primary tumor size (maximum axial diameter) was measured on the co-registered low-dose CT and recorded. For semi-quantitative analysis, SUV_max_ (normalized by body weight), was calculated as the highest tumor voxel value. To harmonize SUV values obtained by two different PET systems, the EQ∙PET reference-based quantification technology was applied [19]. The SUV of the tumor relative to the maximal spleen uptake (for ^68^Ga-DOTA-SSA) or the maximal liver uptake (for ^18^F-FDG) was calculated by dividing the tumor SUV_max_ by the spleen SUV_max_ (SUV_T/S_) or liver SUV_max_ (SUV_T/L_), respectively [20]. To limit the influence of the use of two different peptides in semi-quantitative analysis, possible variation in splenic uptake (SUVs) between the two ^68^Ga-peptides was assessed. Tumor SUV_max_ values obtained with ^68^Ga-DOTANOC and ^68^Ga-DOTATOC were also compared. Furthermore, the ratio between SUV_max_ of SSTR-PET/CT and SUV_max_ of ^18^F-FDG-PET/CT (SUV_max_ ratio) was calculated [20].

### Pathology and immunohistochemistry

Neoplasms were classified in the two categories of lung NET according to the WHO 2021 criteria: typical carcinoid TC/NET-G1 and atypical carcinoid AC/NET-G2 [3]. Histopathological information included pathological stage according to the American Joint Committee on Cancer (AJCC) 8th edition Cancer Staging Manual [21], number of mitoses/2 mm^2^, presence of necrosis and its extension (focal/spotty or extensive), and quantification of intra-tumoral inflammatory component as absent (no lymphocyte detected at × 20 magnification), low (< 10 lymphocytes detected at × 20 magnification field), moderate (10–30 lymphocytes detected at × 20 magnification field), and severe (evident lymphocyte aggregates or > 30 lymphocytes detected at × 20 magnification field).

The following immunohistochemical tests were performed for the study: anti-Ki-67 (MIB1, pre-dilute, Dako, Denmark A/S), anti-somatostatin receptor 2A (SST2) (UMB1, 1:5000, Abcam, USA), anti-somatostatin receptor 5 (SST5) (UMB1, 1:5000, Abcam, USA). The Ki-67 labeling index was expressed as the percentage of positive cells in highest labeling areas. Immunohistochemical stains for SSTR2 and SSTR5 were assessed as proposed by Volante and coworkers [13] with a semi-quantitative scoring system that considers both the subcellular localization and the extent of the staining, as follows: score 0, absence of immunoreactivity; score 1, pure cytoplasmic immunoreactivity, either focal or diffuse; score 2, membranous reactivity in < 50% of tumor cells, irrespective of the presence of cytoplasmic staining; score 3, circumferential membranous reactivity in > 50% of tumor cells, irrespective of the presence of cytoplasmic staining. According to Volante and coworkers, scores 2 and 3 were considered as positive receptor expression and were grouped together; conversely, score 0 and 1 were considered as negative receptor expression [13].

### Statistical analysis

Patients’ characteristics were described as *n* (%) or median (min–max) as appropriate. The normality of continuous variables was assessed through the Shapiro–Wilk test and, in case of normal distribution, mean (with standard deviation) was also provided. Separate descriptive analyses were made according to pathological grading: TC/NET-G1 and AC/NET-G2. The Mann–Whitney test and *χ*^2^ or Fisher’s exact test, as appropriate, were used to detect differences between TC/NET-G1 and AC/NET-G2 in pathological/IHC and PET findings. The Mann–Whitney test for independent variables was also applied to evaluate the impact of the type of peptide (DOTANOC and DOTATOC) on spleen SUV_max_ evaluation. The Kruskal–Wallis test was used to assess the association between SSTR-PET/CT tumor SUV_max_ (a continuous variable not normally distributed) and SSTR2-IHC score (a categorical variable with more than 2 categories), while the non-parametric Spearman’s rank correlation test was applied to correlate SSTR-PET/CT tumor SUV_max_ and proliferation index Ki-67 (both continuous variables not normally distributed). The agreement between the two categorical variables SSTR2-IHC receptor expression (negative/positive) and PET/CT qualitative assessment (negative/positive) was evaluated according to kappa statistics [22]. Statistical analysis and graphs were performed using STATA software (STATA/BE 17.0 for Windows, StataCorp LP, College Station, TX 77,845, USA). Two-sided tests were applied, and the significance level was set at *p* < 0.05. No imputation was carried out for missing data.

## Results

**Fig. 1:**
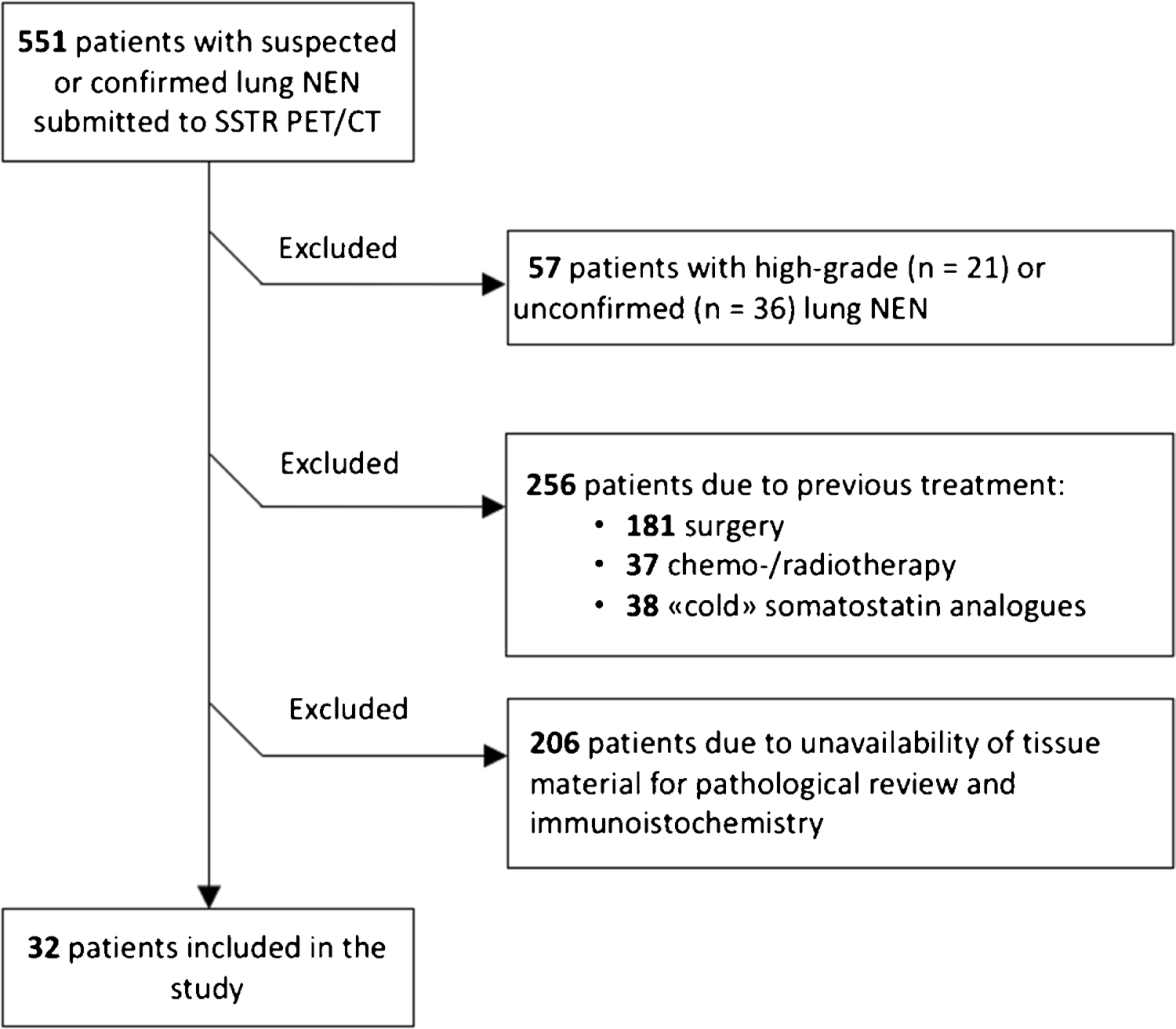
The flowchart of study population

Among 551 patients with suspected or confirmed lung NEN who underwent SSTR-PET/CT at our Center between July 2011 and March 2020, 32 patients fulfilled the inclusion criteria (Fig. 1). ^18^F-FDG-PET/CT was also available for 14 of them. The main clinical, pathological, and immunohistochemical features are reported in Table 1. One patient had histologically confirmed lymph node metastases at the time of surgery, four patients had clinical evidence of distant metastases, and one patient had both lymph node and distant metastases (Table 1). All patients underwent pathological examination by biopsy (eight cases) or surgery (24 cases) at a mean of 31.5 ± 14.5 days from SSTR-PET/CT; pathological and IHC analysis were performed on the lung lesion in all cases. Among all patients, 26 (81.2%) had TC/NET-G1 and 6 (18.8%) AC/NET-G2.

**Table 1:** Clinical and pathological characteristics of 32 patients with lung neuroendocrine tumor

Characteristics	All cases *n* = 32	TC/NET-G1 *n* = 26	AC/NET-G2 *n* = 6	*p* value
Age, years, median (min–max)	62 (29–82)	62.5 (29–82)	62 (56–81)	0.401
Gender				0.242
Male	12 (37.5)	11 (42.3)	1 (16.7)	
Female	20 (62.5)	15 (57.7)	5 (83.3)	
Pathology				
Type of sample				0.117
Biopsy	8 (25.0)	5 (19.2)	3 (50.0)	
Operative tissue	24 (75.0)	21 (80.8)	3 (50.0)	
Mitoses/2 mm^2^, median (min–max)	0.2 (0–3)	0 (0–1.6)	2 (1–3)	**0.0006**
Intra-tumoral inflammatory component	14/31 (45.2)	9/26 (34.6)	5/5 (100)	**0.007**
Immunohistochemistry				
Ki-67%, median (min–max)	4 (1–38)	3 (1–10)	21 (11–38)	**0.0001**
SSTR2				0.258
0	8 (25.0)	5 (19.2)	3 (50.0)	
1	4 (12.5)	3 (11.5)	1 (16.7)	
2	11 (34.4)	9 (34.6)	2 (33.3)	
3	9 (28.1)	9 (34.6)	0 (0)	
Negative receptor expression*	12 (37.5)	8 (30.8)	4 (66.7)	0.102
Positive receptor expression**	20 (62.5)	18 (69.2)	2 (33.3)	
SSTR5				0.905
0	24/31 (77.4)	20/26 (76.9)	4/5 (80.0)	
1	1/31 (3.2)	1/26 (3.8)	0/5 (0)	
2	6/31 (19.4)	5/26 (19.2)	1/5 (20.0)	
3	0/31 (0)	0/26 (0)	0/5 (0.0)	
Negative receptor expression*	25/31 (80.6)	21/26 (80.8)	4/5 (80.0)	0.968
Positive receptor expression**	6/31 (19.4)	5/26 (19.2)	1/5 (20.0)	
Disease staging at diagnosis				
LN metastases°	1 (3.1)	0 (0)	1 (16.7)	0.188
Distant metastases§	4 (12.4)	2 (7.7)	2 (33.3)	0.087
LN° and distant§ metastases	1 (3.1)	1 (3.8)	0 (0)	1

Results are presented as *n* (%) except where indicated. *p* values were calculated with Pearson’s chi-square or Fisher’s exact test for categorical variables and with *t* Student’s or Mann–Whitney *U* test for continuous variables as appropriate. Bold font highlights statistically significant difference

*NET* neuroendocrine tumor, *TC* typical carcinoid, *AC* atypical carcinoid, *SSTR2* somatostatin receptor type 2, *SSTR5* somatostatin receptor type 5, *LN* lymph node

^*^Including scores 0 and 1

^**^Including scores 2 and 3

°Hilar/mediastinal nodal involvement at pathology

^§^At diagnostic imaging

### Pathology

Necrosis was focal/spotty in 3 of 5 AC/NET-G2 (missing data in one case of AC/NET-G2); no samples with extensive necrosis were found. The number of mitoses/2 mm^2^ was significantly higher in AC/NET-G2 (*p* = 0.0006); when present (45.2% of samples), the intra-tumoral inflammatory component was low and more frequent in AC/NET-G2 (*p* = 0.007) (Table 1).

### Immunohistochemistry

Data regarding SSTR2 and SSTR5 receptor expression are reported in Table 1. Overall, when considering scores 2 and 3 as positive receptor expression, SSTR2 was detected in 20/32 samples (62.5%): 18/26 (69.2%) TC/NET-G1 (equal distribution of scores 2 and 3), and 2/6 (33.3%) AC/NET-G2 (all score 2) with no significant difference. SSTR5 (assessed in 31 samples) was detected in 6 samples (19.4%): 5/26 (19.2%) TC/NET-G1 and 1/5 (20.0%) AC/NET-G2 (score 2 in all positive samples) with no significant difference. All samples with a positive SSTR5 expression were also positive for SSTR2. When correlated with disease staging, SSTR2-IHC was positive in 5 of 6 patients (83.3%) with lymph node and/or distant metastases and in 15/26 patients (57.7%) with no lymph node or distant metastases, with no significant difference (*p* = 0.242); SSTR5-IHC (available in 31 patients) was positive in 1 of 6 patients (16.7%) with lymph node and/or distant metastases and in 5/25 patients (20.0%) with no lymph node or distant metastases, with no significant difference (*p* = 0.853). Median Ki-67 was 4% (range 1–38%) (Table 1). A significant difference in Ki-67 expression was found between the two histotypes, with higher values of positivity in AC/NET-G2 (*p* = 0.0001).

### PET/CT evaluation

#### Qualitative analysis

Considering the overall patient series, SSTR-PET/CT was positive (including Krenning scores 1–4) in 28 of 32 cases (87.5%), and ^18^F-FDG-PET/CT in 10/14 (71.4%) (Table 2) with no significant difference (*p* = 0.186). When separately analyzing low- and intermediate-grade tumors, the detection rate of SSTR-PET/CT was 23/26 (88.5%) for TC/NET-G1 and 5/6 (83.3%) for AC/NET-G2, with no significant difference (*p* = 0.732). The detection rate of ^18^F-FDG-PET/CT was 7/11 (63.6%) for TC/NET-G1 and 3/3 (100%) for AC/NET-G2, with no significant difference (*p* = 0.505). A significant difference in tumor size measured at co-registered low dose CT was found, with larger sizes for AC/NET-G2 (*p* = 0.006) (Table 2).

**Table 2:** PET/CT characteristics of 32 low-intermediate grade lung neuroendocrine tumors according to the histotype

Characteristics	All cases *n* = 32	TC/NET-G1 *n* = 26	AC/NET-G2 *n* = 6	*p* value
PET/CT findings				
Maximum tumor diameter, mm*	19 (6–100)	17 (6–36)	61.5 (15–100)	**0.006**
SSTR-PET/CT				
Peptide				0.461
DOTANOC	15 (46.9)	13 (50)	2 (33.3)	
DOTATOC	17 (53.1)	13 (50)	4 (66.7)	
Qualitative assessment				0.732
Negative	4 (12.5)	3 (11.5)	1 (16.7)	
Positive	28 (87.5)	23 (88.5)	5 (83.3)	
Krenning score				0.950
0	4 (12.5)	3 (11.5)	1 (16.7)	
1	12 (37.5)	10 (38.5)	2 (33.3)	
2	2 (6.3)	2 (7.7)	0 (0)	
3	10 (31.3)	8 (30.8)	2 (33.3)	
4	4 (12.5)	3 (11.5)	1 (16.7)	
Tumor SUV_max_	4.4 (0.6–70.3)	4.4 (0.6–70.3)	6.3 (1.2–68.5)	0.809
Spleen SUV_max_	28.3 (6.7–44.2)	28.8 (14.1–44.2)	27.7 (6.7–41.9)	0.981
Tumor SUV_max_/Spleen SUV_max_	0.2 (0.02–2.76)	0.15 (0.02–1.96)	0.27 (0.04–2.76)	0.772
^18^F-FDG PET/CT				
Number of patients	14	11	3	
Qualitative assessment				0.505
Negative	4 (28.6)	4 (36.4)	0 (0)	
Positive	10 (71.4)	7 (63.6)	3 (100)	
Tumor SUV_max_	2.5 (1.5–10.9)	2.1 (1.5–5.8)	7.8 (3.3–10.9)	**0.024**
Liver SUV_max_	3 (2–5.4)	2.9 (2–5.4)	3.6 (3–3.9)	0.184
Tumor SUV_max_/Liver SUV_max_	0.84 (0.3–2.78)	0.78 (0.3–1.88)	2.16 (1.1–2.78)	**0.024**
SSTR-PET/CT—^18^F-FDG PET/CT SUV_max_ ratio	1.35 (0.37–20.78)	1.73 (0.4–20.78)	0.37 (0.37–0.9)	**0.016**

Results are presented as n (%) or median (min–max) as appropriate. *p* values were calculated with Pearson’s chi-square or Fisher’s exact test as appropriate for categorical variables and with Mann–Whitney *U* test for continuous variables. Bold font highlights statistically significant difference

*PET* positron emission tomography, *CT* computed tomography, *SUV* standardized uptake value, *SSTR* somatostatin receptor, ^*18*^*F-FDG* ^18^F-fluorodeoxyglucose

^*^At low-dose CT

#### Semi-quantitative analysis

The analysis of the spleen SUV_max_ values showed a median value of 27.3 (range 14.1–41.4) for ^68^Ga-DOTANOC (15 patients) and 28.9 (range 6.7–44.2) for ^68^Ga-DOTATOC (17 patients), with no statistically significant difference between the two peptides (*p* = 0.395). The analysis of the tumor SUV_max_ values showed a median value of 4.3 (range 0.6–25.4) for ^68^Ga-DOTANOC (15 patients) and 4.6 (range 1.2–70.3) for ^68^Ga-DOTATOC (17 patients), with no statistically significant difference (*p* = 0.356). No significant difference was found between TC/NET-G1 and AC/NET-G2 according to PET parameters (tumor SUV_max_ and SUV_T/S_). Higher values of tumor ^18^F-FDG SUV_max_ and tumor FDG SUV_T/L_ were observed in AC/NET-G2 compared to TC/NET-G1 (*p* = 0.024). Finally, the SUV_max_ ratio between ^68^Ga-DOTA-SSA-PET/CT and ^18^F-FDG-PET/CT showed higher values in TC/NET-G1 (*p* = 0.024) (Table 2).

#### Correlation of PET/CT findings with immunohistochemistry

**Fig. 2:**
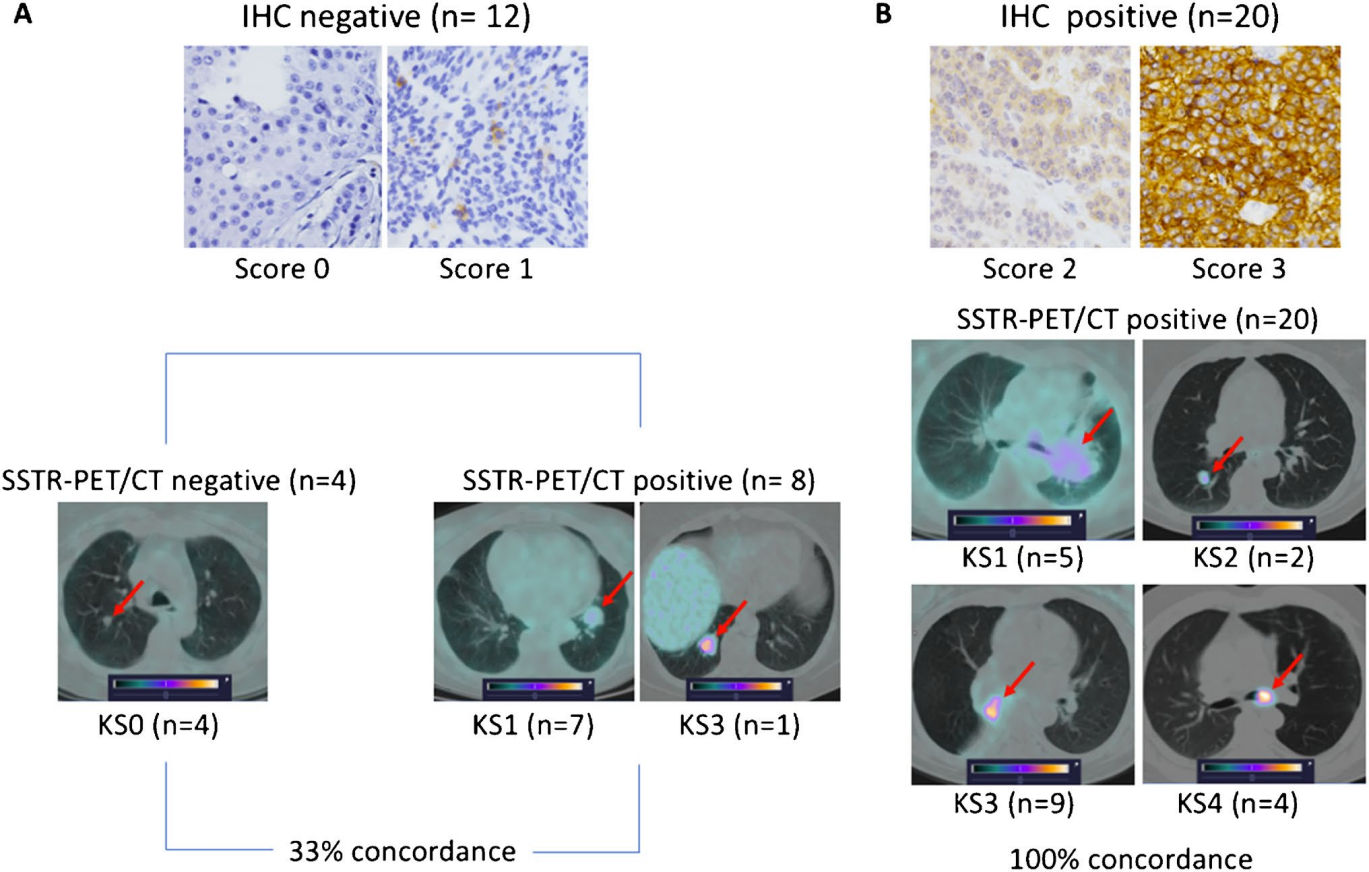
Concordant and discordant findings of pathological and functional assessments with representative images of SSTR2-IHC and SSTR-PET/CT (fused transaxial images) corresponding to the various IHC scores and Krenning scores (KS), respectively; arrows indicate lung lesions. **A** Patients with negative SSTR2-IHC (score 0 and 1) and **B** patients with positive SSTR2-IHC (score 2 and 3)

**Fig. 3:**
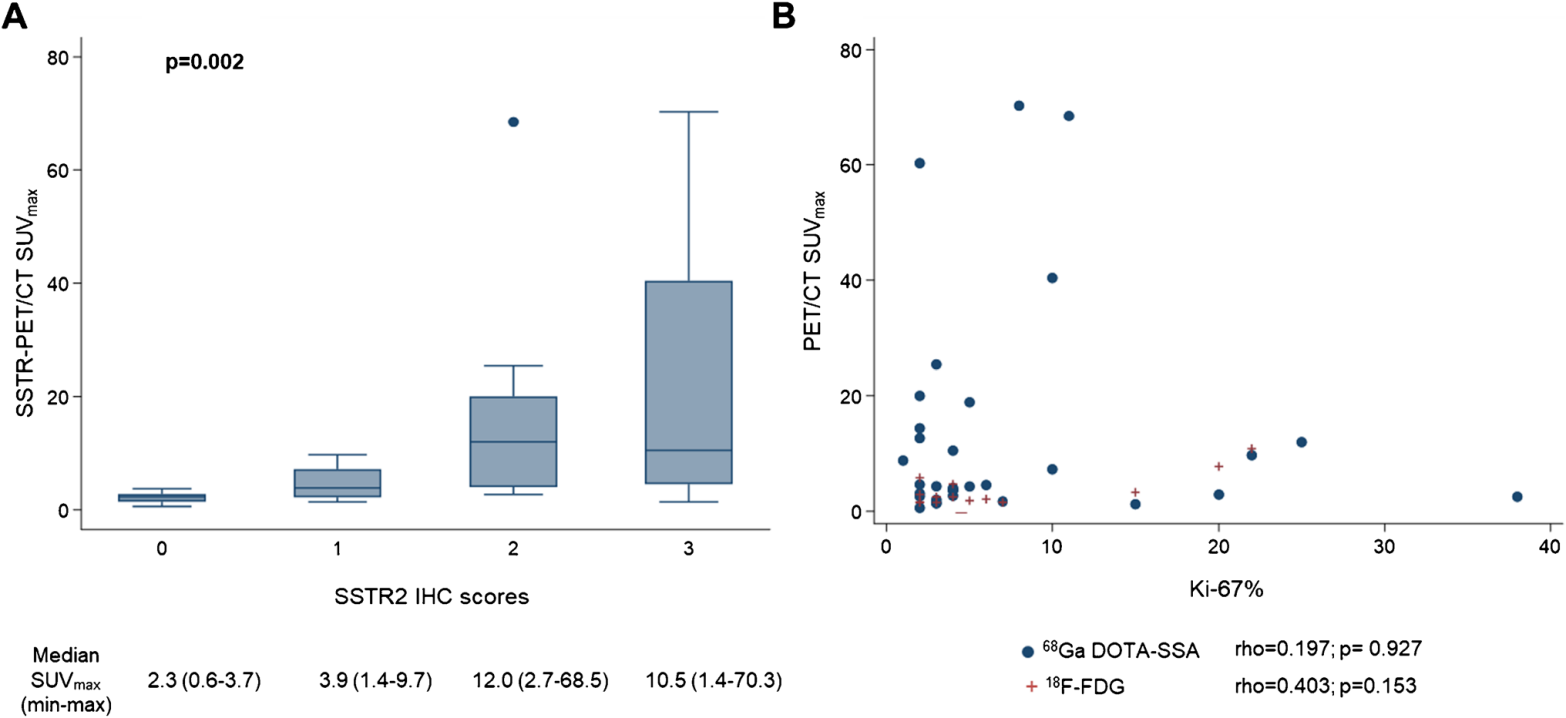
**A** Box plot of SSTR-PET/CT SUV_max_ by SSTR2-IHC scores ranging from 0 to 3. The boxes indicate medians with upper (Q3) and lower quartiles (Q1); the upper and lower bars define values between Q3 − Q3 + 3/2(Q3 − Q1) and Q1–3/2(Q3 − Q1) − Q1, respectively; dots indicate outliers. *p* value was evaluated with two-sided Kruskal–Wallis test. **B** Correlation between SSTR-PET/CT SUV_max_ (blue dots) and ^18^F-FDG-PET/CT SUV_max_ (red crosses) and proliferation index Ki-67 in the overall series. Coefficients of correlation (rho) and *p* values were calculated with two-sided Spearman’s rank correlation test

**Fig. 4:**
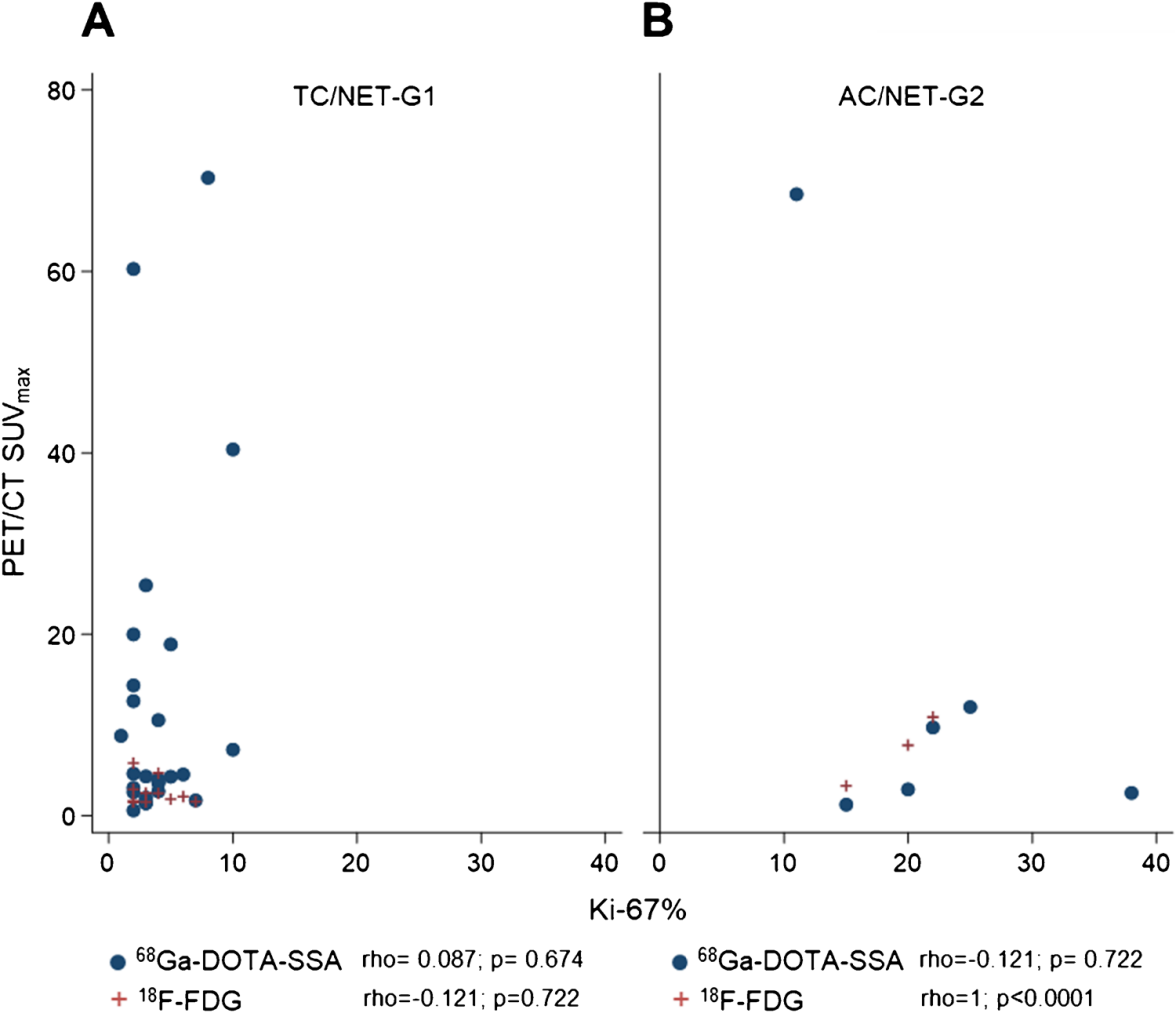
Correlation between SSTR/PET/CT SUV_max_ (blue dots) and ^18^F-FDG-PET/CT SUV_max_ (red crosses) and proliferation index Ki-67 in typical carcinoids (TC/NET-G1) (**A**) and atypical carcinoids (AC/NET-G2) (**B**). Coefficients of correlation (rho) and *p* values were calculated with two-sided Spearman’s rank correlation test

At qualitative assessment, a concordance between SSTR2-IHC and SSTR-PET/CT was found in 24/32 cases (75.0%, *p* = 0.003): 20 were concordantly positive and 4 were concordantly negative. In detail, for positive IHC (scores 2 and 3), 100% concordance with SSTR-PET/CT (both positive) was observed; for negative IHC (score 0 and 1), 33.3% concordance (both negative) was observed (Table 3; Fig. 2). In eight cases, IHC was negative (three biopsies and five surgical samples) while SSTR-PET/CT was positive with tumor uptake lower than liver uptake in seven of these cases (4 TC/NET-G1 and 3 AC/NET-G2) and high tracer uptake (Krenning score = 3) in one AC/NET-G2 displaying SSTR2-IHC score 1. In these eight cases, the median SUV_max_ was 3.0 (range 2.0–9.7). No case with positive IHC and negative SSTR-PET/CT was observed. Figure 3A shows the correlation between SSTR2-IHC scores and SSTR-PET/CT SUV_max_ with a significant difference among the SUV_max_ values for different IHC scores (*p* = 0.002). Figure 3B shows the correlation between ^68^Ga-DOTA-SSA and ^18^F-FDG SUV_max_ values and the proliferation index Ki-67; no significant difference was found. When separately analyzing low- and intermediate-grade tumors, a correlation of strong statistical significance between SUV_max_ and Ki-67 was found for ^18^F-FDG only in lung AC/NET-G2 (rho = 1; *p* < 0.0001), even though the patients observed were only three (Fig. 4).

**Table 3:** Concordance between SSTR-PET/CT qualitative assessment and SSTR2 immunohistochemistry

	Concordant findings			Discordant findings		
	N. patients	IHC +	IHC-	N. patients	IHC +	IHC-
		PET +	PET-		PET-	PET +
TC/NET G1	21	18	3	5	0	5
AC/NET G2	3	2	1	3	0	3
TOTAL	24	20	4	8	0	8

*IHC* immunohistochemistry, *PET* positron emission tomography

## Discussion

In this study, we found an overall good agreement (75.0%, *p* = 0.003) between in vitro SSTR expression and tumor uptake of ^68^Ga-DOTA-SSA at qualitative assessment in a retrospective series of patients with lung NET. For positive SSTR expression, 100% concordance was found. Similarly, higher SUV_max_ values corresponded to membranous staining, which indicates positive receptor expression [13]. These findings may impact on clinical practice in cases where SSTR-PET/CT is not available for preoperative and staging purposes. In these cases, the demonstration of membranous staining in NET biopsy may surrogate SSTR-PET/CT for treatment with “cold” or radiolabeled SSA [14, 23–25]. Indeed, ^68^Ga-DOTA-SSA-PET/CT, showing high affinity for SSTR2 (higher binding affinity of the analog DOTATATE followed by DOTANOC and then by DOTATOC) and, with some ligands, also for SSTR3 (DOTANOC) and/or SSTR5 (DOTANOC and DOTATOC), is considered the most reliable method for in vivo assessment of somatostatin receptor status [26]. It provides information on the presence of SSTR and its affinity for the radio-ligand and allows tumor localization with high sensitivity. Moreover, the intensity of tracer uptake is used to tailor treatment with SSA and select patients suitable for peptide receptor radionuclide therapy [23–25].

All patients included in the present cohort had a well-differentiated lung NET, a finding that is a consequence of the retrospective nature of our study as well as the inclusion criteria. Indeed, patients with poorly differentiated/high-grade lung NEN are rarely submitted to PET/CT studies for staging purposes. Among lung NET patients, we found an 81% prevalence of TC/NET-G1, which is in line with literature data [4].

For in vitro studies, we applied the scoring system proposed by Volante and coworkers [13] which evaluates the subcellular localization (membranous or cytoplasmatic) and the extent of staining. Consistent with previous data obtained in a larger population, SSTR2 was the predominantly expressed receptor subtype with positive findings in 62.5% of samples [27]. SSTR5 was poorly represented with a positive rate of 19.4%, not improving the overall concordance between in vitro and in vivo results. Based on this, further considerations on our findings are essentially based on SSTR2 expression. Scores 2–3 were predominantly detected in TC/NET-G1 (69% vs 33% of AC/NET-G2), even though the difference was not statistically significant, likely due to the few samples of AC/NET-G2 analyzed. In this histotype, score 3 indicating the highest receptor density was never observed in the present series. Among all patients studied, 6 presented lymph node (2 cases) and/or distant metastases (5 cases). IHC for SSTRs was done on the primary site, and it is known to be consistent in primary and metastatic deposits of lung NEN [27]. Even though we observed a higher rate of positive SSTR2-IHC in the metastatic group (83.3%) compared to non-metastatic NENs (57.7%), the difference was not statistically significant, probably due to the small number of patients included. In the study by Righi and coworkers, SSTR2A was overexpressed in metastatic TCs when compared to clinically benign TCs, thus suggesting that SSTR2A status is involved in the metastatic propensity of lung carcinoids [28].

The detection rate of SSTR-PET/CT was 88.5% for TC/NET-G1 and 83.3% for AC/NET-G2, with no significant difference. Therefore, a little amount of SSTR-negative tumors belonged to both histotypes; in other words, SSTR in vivo expression was independent from tumor grade in lung NET. Even when analyzing semi-quantitative data, no significant difference was found between TC/NET-G1 and AC/NET-G2 according to SSTR-PET parameters (tumor SUV_max_ and SUV_T/S_). These observations are in line to what found in corresponding tissue samples at IHC.

We did not find any case of positive IHC and negative SSTR-PET/CT, thus confirming the role of a reference method assigned to functional imaging with radio-receptor PET/CT. Conversely, Papotti et al. and Volante et al. reported cases of positive IHC and negative radio-receptor scintigraphy, a technique that is characterized by lower spatial resolution and lower sensitivity than PET/CT imaging [5, 13]. Moreover, the binding affinity for SSTR2 and SSTR5 of the radiolabeled SSA is significantly affected by structural changes of the octapeptide as well as the choice of chelator (DTPA vs DOTA) or metal used for labeling, with a clear advantage for ^68^Ga-DOTA-labeled compounds over ^111^In-DTPA-octreotide in terms of binding affinity and consequently of imaging findings [29].

As for the IHC negative cases of the present series, only 33.3% concordance was found since SSTR-PET/CT was positive in eight cases, even though with low-grade uptake (Krenning score = 1) in all but one of them. Similar discrepancies have been previously observed, even though in mixed series of NEN patients studied by PET/CT [15] or in patients with lung NEN submitted to ^111^In-DTPA-octreotide scintigraphy [5, 13]. In our series, the visualization (although with low uptake) of a lung lesion with negative SSTR2-IHC could be partly explained by the binding affinity of ^68^Ga-DOTATOC and ^68^Ga-DOTANOC to SSTR2, higher than that of Octreoscan® [29]. In any case, current imaging selection criteria do not consider peptide receptor radionuclide therapy for lesions showing no or low uptake, so most negative cases for SSTR at IHC should remain unsuitable (seven out of eight in this series) for peptide receptor radionuclide therapy despite slightly in vivo positivity.

In addition to neuroendocrine tumor cells, other non-neoplastic cells such as lymphoid and endothelial cells express SSTRs [30, 31]. As functional radio-receptor imaging cannot define the cellular receptor localization, we quantified the intra-tumoral inflammatory component on tissue samples as potential source of positive SSTR-PET/CT imaging. When present (45.2% of samples), the intra-tumoral inflammatory component was low, making it unlikely to be the determinant of radioligand uptake. Conversely, the discordant finding of SSTR expression in vivo vs in vitro could be explained by SSTR expression levels below the detection limit of IHC. On this line, the low values of SUV_max_ observed in discrepant cases would support this potential explanation.

A relevant observation of our study was that in vitro SSTR scores and SUV values at SSTR-PET/CT proceeded at the same rate. Cytoplasmatic staining, indicating negative receptor expression, matched low SUV values, and membranous staining, indicating positive receptor expression, matched higher SUV values (*p* = 0.002) [13]. Therefore, SUV values can be used as a parameter of SSTR2 density. Miederer and coworkers in a mixed series of NEN [15] and Kaemmerer and coworkers in gastro-entero-pancreatic NETs reported a similar highly significant correlation [32]. However, some discrepant results were also observed in our series, as one of 12 tumors with IHC scores 0–1 showed high SUV values (9.7 in one AC/NET-G2) and 3 of 20 tumors with IHC scored 2–3 showed low SUV values (< 4.0, all TC/NET-G1). These discrepancies on the tissue side may reflect non-standard tissue handling for histology processing with resulting poor SSTR preservation, and on the in vivo side, undetermined reduced SSTR expression status or SSTR blockade.

Besides analyzing the receptor profile, we also studied proliferative activity by Ki-67 staining, which, as expected, proved to be an important discriminant factor between TC/NET-G1 and AC/NET-G2, with higher values of positivity in AC/NET-G2 (*p* = 0.0001). Parallel to this observation, a correlation of strong statistical significance between ^18^F-FDG-SUV_max_ (available in 14 patients only) and Ki-67 was found in lung AC/NET-G2 (*p* < 0.0001), even though the calculation could be affected by the small number of patients studied with ^18^F-FDG-PET/CT. Conversely, we did not find any correlation between SUV_max_ at SSTR-PET/CT and Ki-67 values, both in the overall series and when analyzing separately TC/NET-G1 and AC/NET-G2. These findings are in agreement with those reported by Campana and coworkers in a mixed series of NET patients studied with ^68^Ga-DOTANOC [33].

In line with our previous study, the use of SUV_max_ ratio between ^68^Ga-DOTA-peptides and ^18^F-FDG-PET/CT allowed the distinction between TC/NET-G1 and AC/NET-G2 in those lung lesions that are visualized by both tracers. This could be a valid help in surgical management, potentially influencing the extension of parenchymal resection and lymph node dissection, considering that the differentiation between the two histotypes is rarely feasible through pre-operative biopsies [20].

This study has some limitations. First is its retrospective nature. Second, two different SSA were used as radio-ligand, with different affinities to SSTR. To assess the influence on the pharmacokinetics of the two different peptides, we calculated possible variations in splenic uptake; no significant differences were observed. As in a previous multicenter study, we chose the spleen as reference, because it is a healthy organ showing high physiologic uptake of radio-peptides that is homogeneous throughout the splenic parenchyma, and is due to high expression of SSTR [20]. Third, tissue handling though following rigid standard procedures may slightly vary between cases, with improper receptor antigen preservation and poor resulting IHC. Finally, we did not investigate the prognostic value of SSTR2-IHC and SSTR-PET/CT findings since the goal of the original study was to assess the potential role of IHC in predicting in vivo SSTR expression. A long-term analysis of clinical outcome is needed to clarify whether SSTR2-IHC and SSTR-PET/CT findings are predictors of patient outcome, able to stratify lung NET patients with poor prognosis. Nonetheless, this study was performed in a highly selected and well-annotated series of lung NET, while available literature data are relatively old and not specifically targeting lung NET [5, 13, 15, 32].

In conclusion, information on comparative SSTR expression in vitro and in vivo in patients with lung NET is limited. This retrospective study showed an overall good agreement between SSTR2-IHC on tissue and tumor uptake at ^68^Ga-SSA-PET/CT in patients with lung NET. PET/CT with ^68^Ga-SSA was confirmed to be the most reliable method for in vivo assessment of receptor status. In clinical practice, SSTR-PET/CT SUV_max_ values can be used as a parameter of SSTR2 density. Membranous staining in tissue samples is feasible and informative for follow-up studies with SSTR-PET/CT and potentially for treatment with “cold” or radiolabeled SSA analogs. Within the limits imposed by the relatively small cohort here reported, our data suggest that SSTR2-IHC may surrogate SSTR-PET/CT in selected lung NET patients for effective clinical decision making when SSTR-PET/CT is not available or where financial constraints limits the access to in vivo SSTR assessment.

## Data Availability

The datasets generated during and/or analyzed during the current study are available from the corresponding author on reasonable request.
