# Correction to: Correlation of somatostatin receptor PET/CT imaging features and immunohistochemistry in neuroendocrine tumors of the lung: a retrospective observational study

**DOI:** 10.1007/s00259-022-05877-8

**Published:** 2022-06-14

**Authors:** Vittoria Rufini, Margherita Lorusso, Frediano Inzani, Tina Pasciuto, Elizabeth Katherine Anna Triumbari, Lucia Rosalba Grillo, Filippo Lococo, Stefano Margaritora, Edoardo Pescarmona, Guido Rindi

**Affiliations:** 1 Section of Nuclear Medicine, University Department of Radiological Sciences and Hematology, Università Cattolica del Sacro Cuore, Rome, Italy; 2 Unit of Nuclear Medicine, Fondazione Policlinico Universitario A. Gemelli IRCCS, Rome, Italy; 3 ENETS Center of Excellence for the Diagnosis and Cure of Neuroendocrine Tumors, Rome, Italy; 4 PET/CT Center, Fondazione Policlinico Universitario Agostino Gemelli IRCCS, Rome, Italy; 5 Unit of Pathology, Department of Woman and Child Health and Public Health, Fondazione Policlinico Universitario A. Gemelli IRCCS, Rome, Italy; 6 Research Core Facility Data Collection G‑STeP, Fondazione Policlinico Universitario A. Gemelli IRCCS, Rome, Italy; 7 Pathology Unit, San Camillo-Forlanini Hospitals, Rome, Italy; 8 Section of Thoracic Surgery, Department of Translational Medicine and Surgery, Università Cattolica del Sacro Cuore, Rome, Italy; 9 Unit of Thoracic Surgery, Fondazione Policlinico Universitario A. Gemelli IRCCS, Rome, Italy; 10 Pathology Unit, ‘Regina Elena’ National Cancer Institute IRCCS, Rome, Italy; 11 Section of Pathology, Department of Woman and Child Health and Public Health, Università Cattolica del Sacro Cuore, Rome, Italy

**Correction to: European Journal of Nuclear Medicine and Molecular Imaging**

**https://doi.org/10.1007/s00259-022-05848-z**

The authors regret that the name of one of their co-authors appeared incorrect in the original article.

It is now corrected in this erratum article.

